# Mutation analysis of the TNFAIP3 in A20 haploinsufficiency: A case report

**DOI:** 10.1097/MD.0000000000025954

**Published:** 2021-05-21

**Authors:** Mei Yan, Danlu Li, Shakan Aknai, Hongtao Zhu, Mayila Abudureyim

**Affiliations:** The First Affiliated Hospital of Xinjiang Medical University, Urumchi, Xinjiang, China.

**Keywords:** A20, TNFAIP3, haploinsufficiency

## Abstract

**Introduction:**

Haploinsufficiency of A20 (HA20) is a novel genetic disease presented by Zhou et al in 2016. A20 is a protein encoded by TNFAIP3. Loss-of-function mutation in TNFAIP3 will trigger a new autoinflammatory disease: HA20. HA20-affected patients may develop a wide range of clinical manifestations, such as Behcet disease, rheumatoid arthritis, rheumatic fever, juvenile idiopathic arthritis, and systemic lupus erythematosus. HA20 is rarely reported, thus remaining far from thoroughly understood. Sixty-one cases of HA20 have been reported worldwide, among which 29 cases were diagnosed with Behcet disease ultimately. Moreover, 3 cases have been reported in China, which was the first report of HA20 characterized by Behcet disease. A comprehensive understanding of the pathogenic genes of HA20 could help us apply targeted therapy as soon as possible to improve patients’ survival rates.

**Patient concerns:**

A 2-year-old 3-month-old child was presented to our hospital with recurrent infectious enteritis and stomatitis.

**Diagnosis:**

Genetic mutations were detected immediately, and a novel pathogenic mutation was found in TNFAIP3. A heterozygous mutation (c.436-437deTC) located at TNFAIP3 was confirmed. The present research indicated that the TNFAIP3 mutation of c.436-437deTC (p.L147Qfs^∗^7) accounted for familial Behcet-like autoinflammatory syndrome in the child suffering from HA20, while no variation in this locus was found in her parents.

**Interventions:**

Symptomatic treatments including oral administration of prednisone (12.5 mg/d) and iron supplement were performed, and repeated infection was no longer observed in the child. Pain and activity limitation was found in the knee joints. The treatment regimen was adjusted to oral prednisone (12.5 mg/dose, 2 doses/d) and subcutaneous injection of rhTNFR:Fc (12.5 mg/week).

Outcomes: At the last follow-up, the limbs’ activities were normal, the inflammatory indicators were reduced or within the normal range. The prednisone dose was reduced to 7.5 mg/d, while the dose of rhTNFR:Fc was not changed.

**Conclusion:**

We have identified a novel pathogenic HA20 mutation. In this article, 1 case was analyzed in-depth in terms of clinical manifestations of the patient and new sources of such a novel disease, which might improve our understanding of this disease.

## 1 Introduction

A20 haploinsufficiency (HA20) is a novel genetic disease presented by Zhou et al in 2016.^[1]^ A20 is a protein involved in the immune signal regulation encoded by the TNFAIP3 gene, which serves as a negative regulator in the nuclear factor kappa B (NF-κB) signaling pathway.^[2]^ Loss-of-function mutation in TNFAIP3 may weaken the negative regulation of NF-κB signaling pathway while enhancing the inflammasome activation of the nucleotide-binding domain-like receptor protein, both of which may lead to an overproduction of proinflammatory cytokines, including interleukin (IL)-1β, IL-6, IL-18, and TNF-α.^[3]^ As a result, patients with congenital immune dysfunction may develop various immune disorders.^[1,4,5]^ Currently, relevant cases reported were diagnosed with multiple immune diseases, including Behcet's disease, rheumatic fever, systemic lupus erythematosus, juvenile idiopathic arthritis, synovitis, arthritis, and lymphoma.^[5–8]^ Moreover, for some cases, there remained no definite or multiple clinical diagnoses. These patients may present with various symptoms, including recurrent fever, oral ulcers, genital ulcers, joint pain, polyarthritis, bilateral anterior uveitis, or even no clinical specificity.^[5–8]^ So far, a total of 61 cases from 26 families have been reported, including sporadic cases.^[9]^ Clinical manifestations vary considerably, even within 1 family. In addition, there were 29 cases with Behcet disease from 13 families.^[9]^ In this report, we summarized the gene locus and clinical manifestation of a total of 30 cases, including this case, hoping to provide some information about the diagnosis and treatment of this disease.

## 2 Case report

The patient was a 2-year-old and 3-month-old girl. She was admitted to our hospital complaining of “recurrent fever, diarrhea, and vomiting for more than 2 years.” Her mother had no abnormality during pregnancy and delivery. Three months after birth, the child had a recurrent fever, diarrhea, and vomiting. Each attack lasted for about 1 to 2 weeks, with an interval of 1 to 5 months. So far, 11 attacks have occurred. During the onset of the disease, procalcitonin (PCT), C-reactive (CRP), and white blood cell (WBC) count were increased, and many WBC could be seen in the stool. Therefore, the patient was treated with cephalosporins for about 1 week based on the diagnosis of infectious diarrhea, and her condition had improved. The child was prone to recurrent oral ulcers, and she was diagnosed with iron deficiency anemia previously. Still, her symptoms had not been alleviated after the oral administration of iron dextran, with hemoglobin fluctuation between 80 to 90 g/L. In addition, she was allergic to milk, eggs, mango, seafood, and other foods, which may lead to severe vomiting and diarrhea.

**Figure 1:**
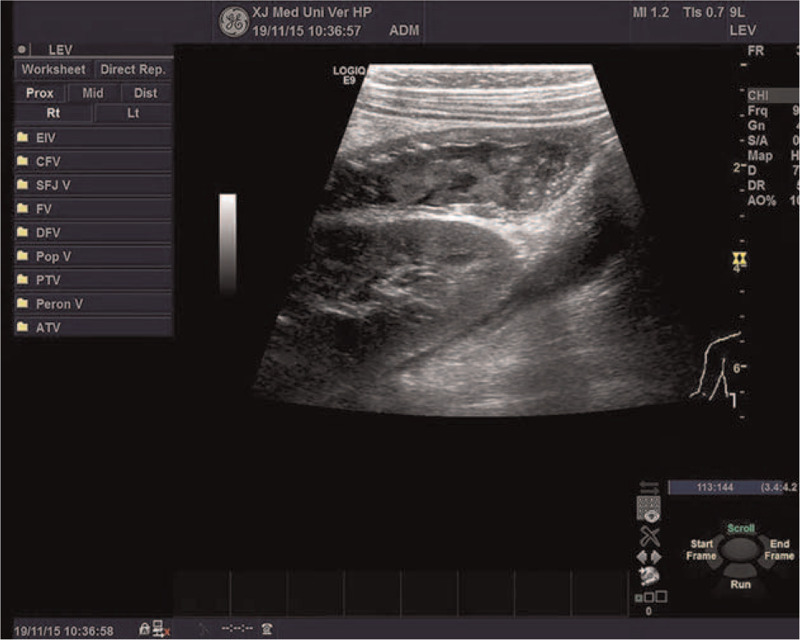
Gastrointestinal ultrasound showed an uneven thickening of the ascending colon and thickening of the surrounding mesentery, about 0.4 cm, suggesting inflammatory changes.

**Figure 2:**
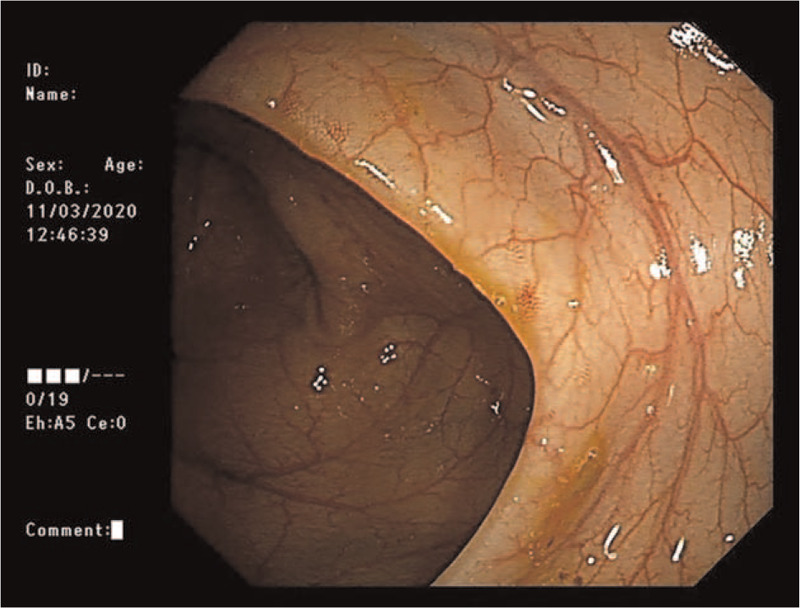
Colonoscopy suggested congestion of the colonic mucosa.

**Figure 3:**
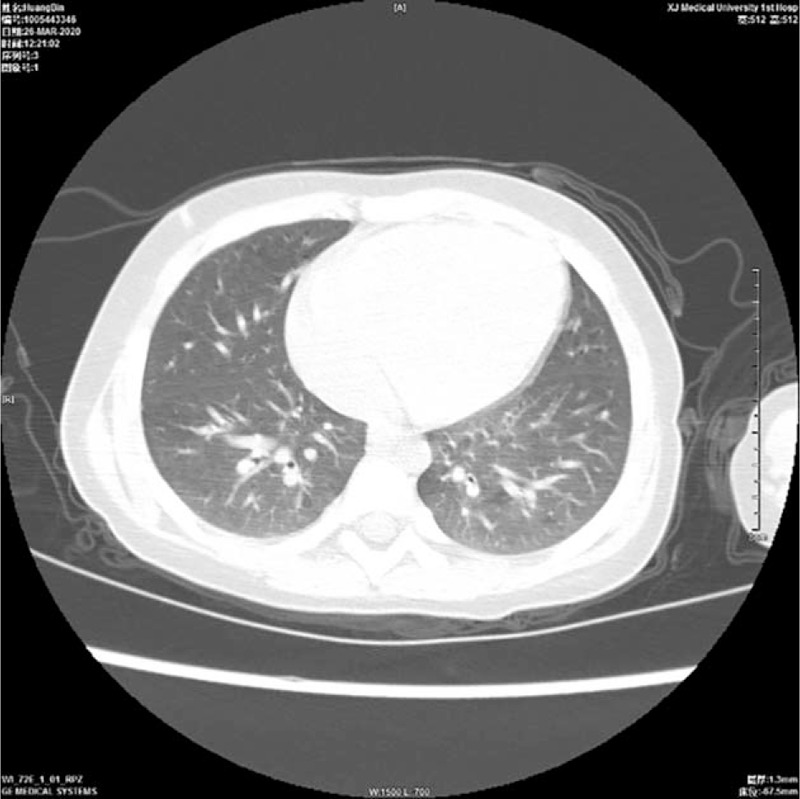
Lung computed tomography indicated that multiple granulomas were present.

Before admission, the child showed vomiting, fever, and diarrhea after eating half of a quail egg, with a maximum body temperature of 39.2 °C, and she had been limping for nearly a week. Her parents denied that she had a history of photophobia, tears, blurred vision, joint pain, and vulvar ulcer. Physical examination showed that the body temperature was 38.4 °C, heart rate was 134 times/min, respiratory rate was 26 times/min, weight was 12 kg, and height was 88 cm. She had facial anemia, no skin rash, no conjunctival congestion, and an oral ulcer of about 0.5 × 0.3 cm. No abnormalities in the heart and lungs were found. The patient had abdominal distention, and her liver could be palpated at 2 cm below the costal margin, and the spleen was palpable 3 cm below the costal margin. The shifting dullness was negative. There was no tenderness and muscle tension. No abnormalities were noted in the vulva and perianal areas. The neurological examination was negative. Supplementary examination: WBC 12.22 × 10^9^/L, NE 8.52 × 10^9^/L, HB 87 g/L; PCT 0.24 ng/mL, CRP 65.5 mg/L, IL-6 65.5 mg/L, VitB12 1118 pmol/L, APTT 69 s, DRJT 942 ng/mL, ESR 58 mm/h, VitB12 118.2 pmol/L, IgE 96.81 IU/ml, natural killer cells 25.2%, total B cells 27.8%, CD4/CD81.0, ANA 1:100, and anticardiolipin antibody 18 RU/mL. Gastrointestinal ultrasound showed an uneven thickening of the ascending colon and thickening of the surrounding mesentery, about 0.4 cm, suggesting inflammatory changes (Fig. 1). Ultrasound showed that there was effusion in the right knee joint cavity and thickened synovium. Colonoscopy suggested congestion of the colonic mucosa (Fig. 2). Lung CT indicated that multiple granulomas were present (Fig. 3).

**Figure 4:**
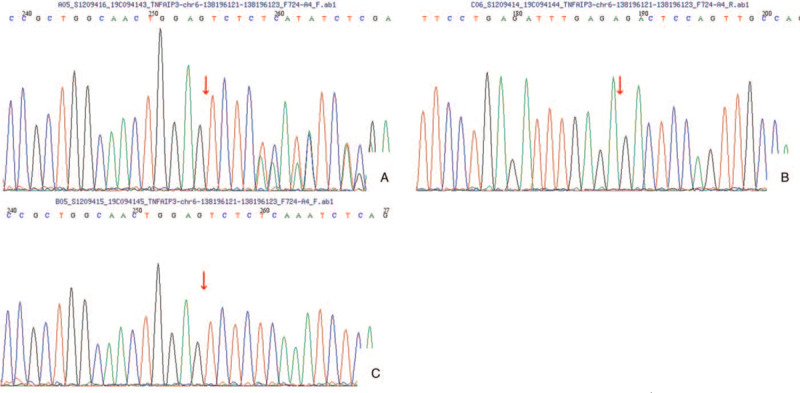
A patient with familial Behcet-like autoinflammatory syndrome (A) had spontaneous mutation c.436_437delTC (p.L147Qfs^∗^7) in the TNFAIP3 gene. There was no mutation at the site of the father (B) and the mother (C).

Since the allergen test indicated multiple allergens and that autoimmune diseases could not be ruled out, whole-exome gene sequencing was carried out from the child's peripheral blood (MyGenostics, Beijing, China). The Sanger sequencing of their parents for pedigree verification is shown in Figure 4. We found that there was a heterozygous mutation (c.436_437deTC) in the TNFAIP3 gene, which resulted in a frameshift mutation of an amino acid (p.L147Qfs^∗^7), and it was a zero effect mutation (frameshift mutation) that may lead to loss of gene function (PVS1). The family verification analysis showed no mutation in the parents; thus, it was a spontaneous mutation (PS2). Based on the analyses above, this variation in the normal population database was a low-frequency variation (PM2), so it was preliminarily determined as pathogenic variation according to the ACMG guidelines.

After the genetic test results were obtained on December 19, 2019, symptomatic treatments including oral administration of prednisone (12.5 mg/d) and iron supplement were performed, and repeated infection was no longer observed in the child. The dose of prednisone was reduced gradually to 10 mg/d. Pain and activity limitation was found in the knee joints in May 2020, and MRI of joints showed synovitis and joint effusion signs. The inflammatory indicators also increased: PCT 16.2 ng/mL, CRP >90 mg/L, and IL-6 218 pg/mL. Therefore, the treatment regimen was adjusted to oral prednisone (12.5 mg/dose, 2 doses/d) and subcutaneous injection of recombinant human type II tumor necrosis factor receptor-antibody Fc fusion protein (rhTNFR:Fc; 12.5 mg/wk). Afterward, the disease was stable, and only gastrointestinal manifestations, including fever and vomiting, were found after intake of cold drink in October 2020, which improved after treatments, including anti-inflammation therapy. Reexamination by colonoscopy showed that the intestinal wall was smooth and with no evident abnormality. Pulmonary CT showed no signs of granuloma. Ultrasound examination of the knee joints showed normal results.

Further reexamination on January 25, 2021, showed that the anemia was corrected, and the treatment with iron supplement was stopped. The limbs’ activities were normal, the inflammatory indicators were ESR 11 mm/h, PCT 0.02 ng/mL, CRP 12 mg/L, and IL-6 8 pg/mL, and TNF was in the normal range. The prednisone dose was reduced to 7.5 mg/d, while the dose of rhTNFR:Fc was not changed.

## 3 Discussion

In affected cases from 6 independent families with familial Behcet-like autoinflammatory syndrome, Zhou et al identified 6 different heterozygous truncating mutations in the TNFAIP3 gene.^[1]^ The mutations in the first 2 families were detected using whole-exome sequencing and confirmed by Sanger sequencing. Three subsequent mutations were observed in 3 of 150 probands with a similar disorder who were directly screened for TNFAIP3 mutations. The sixth mutation was found in one of 768 individuals diagnosed with Behcet disease via targeted sequencing.^[1]^ In vitro studies on functional cellular expression demonstrated that all mutations failed to suppress TNF-induced NF-κB activity, although not in a dominant-negative fashion, which suggested haploinsufficiency as the underlying pathogenic mechanism. Compared with control cells, patient-derived cells exhibited decreased recruitment of TNFAIP3 to the TNFR complex, and increased phosphorylation of IKKA and IKKB and subsequent degradation of IκBα, with nuclear translocation of the NF-κB p65 subunit together with increased expression of NF-κB-mediated proinflammatory cytokines, consistent with activation of the NF-κB pathway. Cells expressing the mutant proteins showed defective removal of lys63-linked ubiquitin from TRAF6, NEMO, and RIP1 after stimulation with TNF, indicating inefficient deubiquitinating. The proinflammatory cytokines’ levels were substantially higher in patients’ serum compared to healthy controls, as robust evidence for enhanced IL1B signaling.^[10,11]^

Cytokines such as TNF exert a profound impact on the function of endothelial cells. For instance, TNF can promote the interaction with leukocytes and induce a procoagulant phenotype. Changes of this nature might play a pivotal role in the proinflammatory effects of TNF. TNF-induced primary response genes were analyzed in human umbilical vein endothelial cells. Of the 6 induced cDNAs identified, 2 encoded paracrine factors (neutrophil chemotactic factor and monocyte chemotactic factor), 1 encoded a membrane receptor for neutrophils (endothelial leukocyte adhesion molecule 1), and 3 encoded undescribed TNF primary response genes hitherto. On exposure of endothelial cells to TNF, there was a rapid and substantial increase in mRNA levels encoding the 6 genes, which were further superinduced by cycloheximide. Thus, these represent primary response genes, as their induction does not depend on protein synthesis. One of the 3 new proteins, designated A20, was found on sequence analysis to code for a novel zinc finger protein.^[12]^

Lu et al generated 2 lines of gene-targeted mice via abrogating either the deubiquitinating activity of A20 (Tnfaip3-OTU mice) or zinc finger-4 of A20 (Tnfaip3-ZF4 mice). Both strains exhibited increased responses to TNF and sensitivity to colitis. The deubiquitinating motif restricted both lys48- and lys63-linked ubiquitination of Rip1. ZF4 was required for the recruitment of A20 to ubiquitinated Rip1. Two mutant A20 proteins complemented each other through dimerization to regulate Rip1 ubiquitination and NF-κB signaling in compound mutant Tnfaip3-OTU/Tnfaip3-ZF4 cells.^[13]^

Lee et al^[3]^ generated A20-deficient mice by targeted disruption. A20 +/− mice appeared normal without evidence of pathology. A20 −/− mice, born from interbred A20 +/− mice in Mendelian ratios, developed runting as early as 1 week old. Mice deficient for A20 developed severe inflammation and cachexia, were hypersensitive to both lipopolysaccharide and TNF, and died prematurely.^[3]^

At present, there were 29 cases of Behcet disease with various clinical symptoms.^[4,5,14–16]^ For example, in this case, the patient had synovitis and pulmonary granuloma in addition to Behcet. In this report, the clinical symptoms, supplementary examination, gene locus, and other characteristics of this case and the 48 cases reported so far are summarized in Table 1.

**Table 1:** Clinical features of patients with TNFAIP3.

Pedigree	Mutation location	Onset age	Sex	Clinical manifestation	Other medical history	Diagnosis	References
Family 1	TNFAIP3	9 yr	M	Fever, perianal, oral, and genital ulcer, rashes, folliculitis	syndrome, 5 relapses after hormone withdrawal	BD, nephrotic syndrome	Shigemura et al^[14]^
		8 yr	F	Oral, genital, intestinal ulcers, rashes, folliculitis	Presence of gastrointestinal symptoms	BD	
		8 yr	F	Fever, oral mucosa, genital ulcer, rashes, folliculitis	\	BD	
		10 yr	F	Oral mucosa, genital ulcer, rashes, folliculitis	\	BD	
Family 2	TNFAIP3 c.252delC p.Trp85GlyfsX11	11 yr	F	Fever, multiple arthritis, oral mucosa, genital ulcer	Presence of gastrointestinal symptoms, Once a month	BD	Kadowaki et al^[4]^
		9 mo	F	Fever, stomatitis	Presence of gastrointestinal symptoms	BD	
Family 3	TNFAIP3 c.728G > A p.Cys243Tyr	17 yr	M	Fever, oral mucosa, genital ulcer	Refractory nephrotic syndrome	BD, nephrotic Syndrome	
		20 yr	F	Fever, oral mucosa, genital ulcer	\	BD	
		Adolescence	F	Fever, oral mucosa, genital ulcer	\	BD	
		Adolescence	F	Fever, oral mucosa, genital ulcer	\	BD	
		12 yr	F	Fever, oral mucosa, genital ulcer	Colonic perforation and pneumocystis pneumonia	BD	
Family 4	TNFAIP3 C.1346delA p.Asn449ThrfsX29	Infancy	F	Fever, abdominal pain, diarrhea, oral mucosa, genital ulcer	Colonic perforation and Pneumocystis pneumonia	BD	
Family 5	TNFAIP3 c.133C > T p.Arg45X Heterozygosity	1 yr	M	Fever, oral, genital, intestinal ulcers	/	BD, intermittent fever	
Family 6	TNFAIP3 p.Leu227^∗^	10 mo	F	Fever, axillary and inguinal lymph nodes, diarrhea, bloody stool, multiple arthritis, oral mucosa, genital ulcer, bilateral anterior uveitis, cataract, glaucoma	Recurrent upper respiratory tract infection in childhood, severe Cushing's syndrome, growth retardation, premature ovarian failure, map tongue	Juvenile idiopathic arthritis, BD	Aeschlimann et al^[5]^
		1 yr	F	Diarrhea, bloody stool, multiple arthritis, oral mucosa, genital ulcer, bilateral anterior uveitis, cataract, glaucoma, adolescent acne, eczema, pityriasis rosea	Mastitis, Cushing's syndrome, map tongue	Juvenile idiopathic arthritis, BD	
		20 yr	F	Multiple arthritis, oral mucosa, genital ulcer, skin abscess of genital area, armpit, and breast; adolescent acne	Recurrent upper respiratory tract infection and radiation-treated goiter in childhood	Rheumatoid, BD	
Family 7	TNFAIP3 p.Phe224Serfs^∗^4	8 yr	F	Multiple arthritis, oral mucosa, genital ulcer, pustular, folliculitis like rash	Membranous nephropathy; irregular menstruation, late menarche (18 years old)	BD, lupus nephritis	
Family 8	TNFAIP3 p.Arg271^∗^	9 mo	M	Fever, diarrhea, bloody stool, arthralgia, oral, genital, intestinal ulcers	Repeated upper respiratory tract infection and pericardial effusion	BD	
		Unknow	M	Oral mucosa, genital ulcer	/	BD	
Family 9	TNFAIP3 p.Tyr306^∗^	4 yr	F	Abdominal pain, foot pain, oral mucosa, genital ulcer	Cerebral palsy associated with recurrent infection, autism, and cerebral palsy	Suspected BD	
		Infancy	F	Abdominal pain, diarrhea, arthralgia, oral mucosa, genital ulcer, severe acne on face, buttock, and groin	Rectal bleeding, repeated infection, dysmenorrhea and hysterectomy	Suspected BD	
Family 10	TNFAIP3 p.Pro268Leufs^∗^19	29 yr	F	Muscle and joint pain, oral mucosa, genital ulcer, acne like rash of limbs	Dental problems	BD	
		15 yr	F	Oral mucosa, genital ulcer	/	Suspected BD	
		13 yr	F	Oral mucosa, genital ulcer	/	Suspected BD	
Family 11	TNFAIP3 p.338X	7 d	M	Fever, abdominal pain, diarrhea, bloody stool, oral, genital, intestinal ulcers, cataract, nonspecific rash	Pulmonary embolism, rheumatoid fibroepithelial polyp, encephalopathy, vertebral compression fracture, hyperglycemia, arterial hypertension	CD, BD	
Family 12	TNFAIP3 c.252delC p.Trp85GlyfsX11	11 yr	F	Fever, arthritis, oral, genital, intestinal ulcers, aquatic keratoderma	hemorrhoids	BD	Ohnishi et al^[15]^
		9 mo	F	Oral, genital, intestinal ulcers	Repeated hematemesis in infancy	BD	
Family 13	TNFAIP3 c.994G > T p.Glu332	6 yr	F	Fever, abdominal pain, hip and joint pain, backache, knee arthritis, intestinal ulcers, unilateral scleritis, severe inflammatory swelling of skin after vaccination	Cough, weakness, anal fissure, thrombophlebitis of lower extremity	BD	Aeschlimann et al^[16]^
		6 mo	F	Fever, abdominal pain, vomit, diarrhea, gastrointestinal hemorrhage, arthritis, intestinal ulcers, pseudofolliculitis, urticaria	Recurrent pharyngitis	BD	
This case	TNFAIP3 c.436_437deTC p.L147Qfs^∗^7 heterozygosity	3 mo	F	Fever, lymphadenopathy, abdominal pain, vomit, diarrhea, synovitis of right knee, splenomegaly, liver enlargement, oral, genital, intestinal ulcers, nonspecific rash	Anemia, multiple allergens, pulmonary granuloma, splenomegaly, hypercoagulable state	BD, synovitis	/

HA20 is a genetic disease about which we are still ill-informed, whose definite treatment and prognosis remained poorly explored. Using second-generation sequencing and according to the ACMG guidelines, the gene locus of this case was newly discovered to account for HA20. In this article, we reported and analyzed 1 case in detail and expected to offer clues for the future diagnosis and treatment of this disease.

## Author contributions

**Conceptualization:** Danlu Li.

**Data curation:** Danlu Li.

**Formal analysis:** Hongtao Zhu.

**Funding acquisition:** Shakan. Aknai.

**Investigation:** Shakan. Aknai.

**Methodology:** Mei Yan.

**Project administration:** Mei Yan.

**Resources:** Danlu Li.

**Software:** Mayila. Abudureyim.

**Supervision:** Mei Yan.

**Validation:** Hongtao Zhu.

**Visualization:** Danlu Li.

**Writing – original draft:** Danlu Li.

**Writing – review & editing:** Danlu Li.
